# Arbovirus, herpesvirus, and enterovirus associated with neurological syndromes in adult patients of a university hospital, 2017-2018

**DOI:** 10.1590/0037-8682-0127-2021

**Published:** 2021-11-12

**Authors:** Lucas Lopes Leon, Rodrigo Gonçalves de Lima, Lídia Cristian Boffi, Raissa Nery Bindilatti, Célia Regina Garlipp, Sandra Cecília Botelho Costa, Sandra Helena Alves Bonon

**Affiliations:** 1 Universidade Estadual de Campinas, Faculdade de Ciências Médicas, Laboratório de Virologia, Campinas, SP, Brasil.; 2 Universidade Estadual de Campinas, Faculdade de Ciências Médicas, Departamento de Patologia Clínica, Campinas, SP, Brasil.

**Keywords:** Arbovirus infections, Central nervous system, Polymerase chain reaction, Viral diseases

## Abstract

**INTRODUCTION:**

Herpesviruses, enteroviruses, and arboviruses are important because of their clinical relevance and ability to cause meningitis, encephalitis, meningoencephalitis, and other diseases. The clinical virology associated with diagnostic technologies can reduce the morbidity and mortality of such neurological manifestations. Here we aimed to identify the genomes of agents that cause neurological syndromes in cerebrospinal fluid (CSF) samples from patients with suspected nervous system infections admitted to the University Hospital of the University of Campinas, São Paulo, Brazil, in 2017-2018.

**METHODS:**

CSF samples collected from adult patients with neurological syndrome symptoms and negative CSF culture results were analyzed using polymerase chain reaction (PCR), reverse transcriptase-PCR, and real-time PCR, and their results were compared with their clinical symptoms. One CSF sample was obtained from each patient.

**RESULTS:**

Viral genomes were detected in 148/420 (35.2%) CSF samples: one of 148 (0.2%) was positive for herpes simplex virus-1; two (0.5%) for herpes simplex virus-2; eight (1.9%) for varicella-zoster virus; four (1%) for Epstein-Barr virus; one (0.2%) for cytomegalovirus; 32 (7.6%) for human herpesvirus-6; 30 (7.1%) for non-polio enterovirus; 67 (16.0%) for dengue virus, three (0.7%) for yellow fever virus, and 21 (5%) for Zika virus.

**CONCLUSIONS:**

The viral genomes were found in 35.2% of all analyzed samples, showing the high prevalence of viruses in the nervous system and the importance of using a nucleic acid amplification test to detect viral agents in CSF samples.

## INTRODUCTION

Acute viral infections of the central nervous system (CNS) may be caused by a large spectrum of viruses. The diagnosis of the etiological agents may be difficult since the symptomatology is diverse and several agents may play a role in neurological outcomes, even ruling out bacterial and fungal origins. Regarding viral infections, arbovirus, non-polio enterovirus (EV), and human herpesvirus (HHV) can have harmful effects, including neurological diseases and death^1^ ^-^ ^3^. 

Populations living in environments with the endemic circulation of these neurotropic viral agents are at constant risk of contracting infections; in these regions, socioeconomic damage is linked to the associated morbidity. A variety of viruses that can invade and cause disease in the nervous system (NS), can be identified in the cerebrospinal fluid (CSF)^1^ ^,^ ^2^ ^,^ ^4^ ^-^ ^6^.

Arboviral infections have become more frequent due to urbanization, deforestation, and other anthropogenic effects. The Zika virus (ZIKV) is notorious due to its range of neurological manifestations in fetuses and newborns, but it has also been associated with outcomes in adults with meningoencephalitis, myelitis, and Guillain-Barré syndrome (GBS). The dengue virus (DENV), yellow fever virus (YFV) are endemic in Brazil and associated with neurological manifestations. EV is strongly associated with viral meningitis and responsible for approximately 90% of all cases worldwide. This viral group is considered the most common cause of aseptic meningitis and one of the most frequent CNS infections. HHVs are often associated with encephalitis. The mortality rate of viral encephalitis is reportedly 3.8-7.4%^1^ ^,^ ^3^ ^,^ ^6^ ^-^ ^14^.

The traditional investigations of CNS infections use culture and focus on searching for other non-viral microorganisms, an investigative gap that should not be overlooked. Negative Gram staining in samples from patients with CNS infection may achieve inconclusive diagnosis ratios in 70% of all cases. In addition, due to some clinical aspects, the diagnosis can be acquired in unspecific terms such as “viral infection” or “bacterial infection”^2^ ^,^ ^15^. 

Polymerase chain reaction (PCR) is widely used for viral identification, and the nucleic acid amplification test (NAAT) is one of the most notorious diagnostic tools used worldwide. The choice of reverse transcription (RT)-PCR, PCR, and quantitative real-time PCR (qPCR) is justified by their successful use, providing fast and reliable identification of neurotropic virus from the CSF without the need for culture, especially in the acute phase, when the viral loads tend to be higher^3^ ^,^ ^13^ ^,^ ^15^. 

Thus, this study aimed to identify the viruses that cause neurological syndromes in adult patients admitted to a university hospital in Campinas, Brazil, using NAAT to identify the possible presence of herpesviruses, enteroviruses, and arboviruses and analyze the viruses: ZIKV, DENV, Chikungunya (CHIKV), YFV, Rocio (ROCV), West Nile Fever (WNV), Ilhéus (ILHV), Aura, St. Louis encephalitis (SLEV), Western equine encephalitis (WEEV), Eastern equine encephalitis (EEEV), Venezuelan equine encephalitis (VEEV) and Mayaro (MAYV), herpes simplex viruses 1 and 2 (HSV-1, HSV-2), and the varicella-zoster virus (VZV), Epstein-Barr virus (EBV), human cytomegalovirus (HCMV), human herpesvirus 6 (HHV-6), and EV. This pioneering study investigated essential data about the viruses that circulate in this metropolis as possible significant patterns of the most important viruses responsible for different clinical manifestations.

## METHODS

This was a descriptive, single-center, cross-sectional study. It included CSF samples from adult patients (one per patient) with viral acute neurological syndrome aged equal to or older than 18 years from January 2017 to July 2018. CSF samples were acquired at the Laboratory of Biological Fluids/Division of Clinical Pathology/University of Campinas. CSF collection was performed in patients from the Hospital de Clínicas. The samples were tested for total protein, glucose, red blood cell (RBC) counts, and white blood cell (WBC) counts. Microbiological tests of fungal and bacterial cultures were performed at the Microbiology Laboratory of the Hospital de Clinicas*.* The etiological agents for all viruses included in this study were not routinely monitored. CSF samples (≥ 400 µL) were sent to the Laboratory of Virology of the School of Medical Sciences/University of Campinas for viral screening using NAAT. All specimens were stored at -80°C.

CSF samples obtained from patients with acute neurological syndrome that tested negative for fungal and bacterial cultures were considered eligible for the study. The samples were collected after consent was received from patients or, when necessary, their legal guardians. The research protocol was designed according to the requirements for research involving human subjects in Brazil. All procedures were performed in accordance with the ethical standards for research involving human beings of the institutional and/or national research committee and the 1964 Helsinki declaration and its later amendments. The research was approved by the Research Ethics Committee (CAAE number 59361816.3.0000.5404).

Nucleic acid extraction and purification were performed using 200 µL of CSF for DNA and 140 µL for RNA according to the protocol of the DNA Biopur Kit (Biometrix, Curitiba, Brazil) and QIAamp Viral RNA Mini Kit (Qiagen, Inc., Valencia, CA), respectively. The extracted and purified products were stored at -80ºC. After the purified RNA was obtained, cDNA was synthesized and reverse-transcribed using a High-Capacity cDNA Reverse Transcription Kit (Applied Biosystems, Foster City, CA, USA), and the cycling conditions were 25°C for 10 min, 37°C for 120 min, and 85°C for 5 min. The eluted product was 60 µL of cDNA, which was stored in a freezer at -80°C. The samples were subjected to PCR of the β2-microglobulin gene to guarantee the quality of the extracted nucleic acid and confirm the absence of PCR inhibitors^16^.

The PCR and nested-PCR (NPCR) reactions were performed in a total volume of 10 µL, containing 0.5 µL of extracted DNA for PCR (and 0.5 µL of the PCR product for NPCR), 5.0 µL of GoTaq® Green (Promega, Winchester, USA), 0.5 µL of each primer, and 3.5 µL of ultrapure water. Electrophoresis was performed using 5.0 µL of the amplified NPCR in a 2% agarose gel, stained with Unisafe Dye (Uniscience, Osasco, Brazil), and subjected to ultraviolet light to visualize the specific DNA bands. The qPCR were performed using the TaqMan™ Fast Advanced (Applied Biosystems), primers and hydrolysis probes (sequences design described elsewhere) synthetized as PrimeTime qPCR Primers (Integrated DNA Technologies Inc., Coralville, IA, USA) and the assays were performed using a StepOnePlus™ Real-Time PCR System (Applied Biosystems) as follows: 95ºC for 20 sec, 40 cycles of 95ºC for 1 sec, and 60ºC for 20 sec. 

NAAT for each specific virus was determined according to a method described elsewhere. 

Regarding herpesviruses, the primer sets were used for each conserved region of HSV-1, HSV-2, VZV, EBV, HCMV, and HHV-6. HHV-DNA was detected in the CSF samples using the NPCR technique, while that for HHV-6 used NPCR and qPCR (qualitative with SYBR green; Thermo Fisher Scientific Baltics, Vilnius, Lithuania)^17^ ^-^ ^23^.

The genus EV was detected according to previously described methodologies using HK-2, HK-3, and HK-10 primers^3^ ^,^ ^18^ ^,^ ^24^. 

The methodology described by Bronzoni et al. was used for the detection of flaviviruses and alphaviruses^3^ ^,^ ^25^ ^,^ ^26^. 

For DENV detection, the methodology described by Huhtamo *et al.* was also used for the qPCR application^27^. 

For CHIKV and ZIKV, the methodology adopted followed the information described by Lanciotti et al.^28^ ^,^ ^29^. 

To avoid contamination, we applied several precautions: use of at least two negative controls to exclude contamination; usage of filter tips for each sample; each phase of the experimental procedure (e.g., extraction of viral nucleic acids) was performed in different rooms; and to guarantee the accuracy of amplification, positive controls were included in each reaction^20^ ^,^ ^30^.

Variables are described using absolute (n) and percentage values and descriptive statistical results, for example, median, mean, standard deviation, and interquartile reference. Fisher’s exact test and the Mann-Whitney U test were used as appropriate. The significance level adopted in this study was 5%.

## RESULTS

CSF samples from 420 patients with suspected CNS infection were included in the study and considered eligible for analysis. All included patients had a suspected CNS infection without positive results of bacterial and fungal detection. A total of 148 specimens were positive for at least one of the viruses investigated in this study. The patients’ characteristics, initial diagnostic hypothesis (IDH), and viral detection results are shown in Table 1. The mean total protein and glucose detected in the CSF in viral PCR negative and positive cases as well as cell count are presented in Table 2. 

**TABLE 1:** Characteristics of 420 Patients with Neurological Syndromes.

	N (%)	Positive	Negative	
	Total N= 420	(n= 148)	(n=272)	P Value^a^
Age, median (range), y	46 (18-92)	47 (18-83)	46 (18-92)	-
Sex				
Female	224 (53.3)	84 (56.7)	140 (51.8)	0.308
Male	196 (46.7)	64 (43.2)	132 (48.5)	
IDH:				
Meningitis	61 (14.5)	24 (5.7)	37 (5.7)	0.472
DC	52 (12.4)	16 (3.8)	36 (3.8)	0.537
IS	36 (8.6)	9 (2.1)	27 (2.1)	0.205
IH	34 (8.1)	11 (2.6)	23 (2.6)	0.852
Encephalitis	27 (6.4)	10 (2.4)	17 (2.4)	0.837
Neuritis	27 (6.4)	13 (3.1)	14 (3.1)	0.151
Seizure	26 (6.2)	7 (1.7)	19 (1.7)	0.405
Headache	25 (6.0)	7 (1.7)	18 (1.7)	0.521
Multiple sclerosis	19 (4.5)	8 (1.9)	11 (1.9)	0.624
Hydrocephalus	15 (3.6)	6 (1.4)	9 (1.4)	0.785
Paralysis	15 (3.6)	5 (1.2)	10 (1.2)	1.000
GBS	14 (3.3)	5 (1.2)	9 (1.2)	1.000
ND	13 (3.1)	3 (0.7)	10 (0.7)	0.557
Dementia	12 (2.9)	3 (0.7)	9 (0.7)	0.552
Myelitis	8 (1.9)	3 (0.7)	5 (0.7)	1.000
Neuropathy	8 (1.9)	4 (1.0)	4 (1.0)	0.460
Behavior disorder	7 (1.7)	3 (0.7)	4 (0.7)	0.701
Meningoencephalitis	7 (1.7)	3 (0.7)	4 (0.7)	0.701
Encephalopathy	5 (1.2)	4 (1.0)	1 (1.0)	0.054
Cerebellar syndrome	5 (1.2)	2 (0.5)	3 (0.5)	1.000
Shunt Derivation	4 (1.0)	2 (0.5)	2 (0.5)	0.616

**IDH:** Initial diagnosis hypothesis; **y:** year old; **DC:** Disorder of consciousness; **IS:** infectious screening/sepsis; **IH:** Intracranial hypertension; **GBS:** Guillain Barré Syndrome; **ND:** Neurodegenerative disease. ^a^P Values indicate differences between positive and negative patients for virus detection. P<0.05 was considered statistically significant.

**TABLE 2:** CSF analysis in Positive and Negative PCR patients with Neurological Syndromes.

	Negative samples (272)			Positive samples (148)			
		IQR			IQR		P Value*
CSF particles	Mean ± SD	Median	25%-75%	Mean ± SD	Median	25%-75%	
Total protein (mg/dl)	56.8±70.9	40	31-57.3	58.7±63.9	43	31-63	0.460
Glucose (mg/dl)	69.4±20.4	63	57-75	69.8±27.2	61.5	55-78.3	0.187
RBC cell/mm³	409.2±4,093.7	7.5	1-47	113.7±263.1	9.5	1-90.3	0.191
WBC cell/mm³	21.5±259.7	2	1-3	61.5±428.6	2	1-5	0.379

**CSF:** Cerebrospinal fluid; **RBC:** Red blood cells; **WBC:** White blood cells; **IQR:** Interquartile reference; **SD:** Standard deviation; *P-value: Mann Whitney test.

Viral genomes were detected in 148/420 (35.2%) of the CSF samples. The most frequently identified nucleic acids in the CSF samples were DENV-positive [67/148 (16.0%)]. A mono-infection, that is, only one virus detected in the same sample, was observed in 128/148 (86.5%) samples, and DENV was the most prevalent, followed by EV, HHV-6, and ZIKV, with 56/148 (37.8%), 23/148 (15.5%), 21/148 (14.9%), and 18/148 (12.2%) samples, respectively. VZV was identified by positive viral PCR in 5/148 (3.4%) samples. EBV was identified in two cases of mono-infection and HCMV and YFV in one case each. 

Codetection, that is, two or more viruses detected in the same CSF sample, occurred in 20/148 (13.5%) samples. Of these 20 samples, 19 (95%) were composed of two viruses identified in the same sample, while one (5%) contained HHV-6+EV+VZV. DENV was the most prevalent virus detected in codetections, identified in 11/20 (55%) of these samples: 5/20 (25%) with HHV-6, 4/20 (20%) with EV, 1/20 with VZV (5%), and 1/20 with ZIKV (5%). HHV-6 was the second most prevalent virus in codetection, being observed in 10/20 (50%) of the codetection cases; in addition to the above described, it was observed with EBV [1/20 (5%)], YFV [1/20 (5%)], and EV [2/20 (10%)] samples. Regarding codetection, there was one sample of each of the following profiles: EBV+YFV, ZIKV+VZV, and ZIKV+EV, representing 5% of the codetection cases.

The prevalence of viruses in the CSF of patients with acute neurological syndromes are reported in Table 3. The general characteristics (age and sex) of the patients in which the viruses were detected, and the positive and negative CSF results and IDH are shown in Table 4.

**TABLE 3:** Prevalence of viruses in CSF of patients with acute neurological syndromes.

Virus	Positive (%)	Negative (%)	Age, median (range), y	Sex	
				Male	Female
HSV-1	1 (0.2)	419 (99.8)	34 (-)	0 (0.0)	1 (100.0)
HSV-2	2 (0.5)	418 (99.5)	26 (18-34)	1 (50.0)	1 (50.0)
VZV	8 (1.9)	412 (98.1)	43 (26-78)	4 (50.0)	4 (50.0)
EBV	4 (1.0)	416 (99.0)	43 (35-52)	1 (25.0)	3 (75.0)
HCMV	1 (0.2)	419 (99.8)	26 (-)	0 (0.0)	1 (100.0)
HHV-6	32 (7.6)	388 (92.4)	52 (18-75)	14 (43.8)	18 (56.3)
EV	30 (7.1)	390 (92.9)	47.5 (18-76)	11 (36.7)	19 (63.3)
DENV	67 (16.0)	353 (84.0)	47 (18-83)	30 (44.8)	37 (55.2)
YFV	3 (0.7)	417 (99.3)	58 (35-72)	1 (33.3)	2 (66.7)
ZIKV	21 (5.0)	399 (95.0)	35 (18-76)	11 (52.4)	10 (47.6)

**CSF:** cerebrospinal fluid; **HSV-1:** Herpes simplex virus-1; **HSV-2:** Herpes simplex virus-2; **VZV:** Varicella-Zoster virus; **EBV:** Epstein-Barr virus; **HCMV:** human cytomegalovirus; **HHV-6:** human herpesvirus 6; **EV:** Non-polio enterovirus; **DENV:** dengue virus; **YFV:** yellow fever virus; **ZIKV:** Zika virus; **NEG:** negative PCR detection; **POS:** positive PCR detection; **y:** years.

**TABLE 4:** Comparison between Positive and Negative CSF results and IDH.

IDH - N	HSV-1	HSV-2	VZV	EBV	HCMV	HHV-6	EV	DENV	YFV	ZIKV
P Value*										
Headache	1	1	-	-	-	1	-	5	-	-
	0.060	0.116	-	-	-	0.710	0.241	0.573	-	-
Dementia	-	-	-	-	-	1	-	1	-	1
	-	-	-	-	-	1.000	-	0.700	-	0.464
Shunt Derivation	-	-	1	-	-	-	-	1	-	-
	-	-	*0.074*	-	-	-	-	0.502	-	-
Behavior disorders	-	-	-	-	-	-	-	3	-	-
	-	-	-	-	-	-	-	*0.084*	-	-
ND	-	-	-	-	-	-	-	3	-	-
	-	-	-	-	-	0.611	0.612	0.445	-	-
Encephalitis	-	-	-	-	-	2	1	4	2	2
	-	-	-	-	-	1.000	0.709	1.000	0.012	0.637
Encephalopathy	-	-	-	-	-	-	1	3	-	-
	-	-	-	-	-	-	0.311	0.031	-	-
Multiple sclerosis	-	-	1	-	-	2	2	4	-	-
	-	-	0.312	-	-	0.648	0.637	0.522	-	-
GBS	-	-	-	-	-	-	3	3	-	1
	-	-	-	-	-	-	*0.070*	0.476	-	0.518
IH	-	-	-	-	-	1	2	6	-	2
	-	-	-	-	-	0.498	1.000	0.807	-	0.683
Hydrocephalus	-	-	-	-	-	3	1	4	-	-
	-	-	-	-	-	*0.098*	1.000	0.275	-	-
Meningitis	-	1	3	1	-	9	4	9	-	2
	-	0.270	*0.095*	0.468	-	0.034	1.000	1.000	-	0.752
Meningoencephalitis	-	-	-	-	-	2	-	2	-	-
	-	-	-	-	-	*0.093*	-	0.310	-	-
Myelitis	-	-	-	1	-	2	1	-	-	-
	-	-	-	*0.074*	-	0.118	0.450	-	-	-
Neuritis	-	-	-	1	-	1	6	1	1	1
	-	-	-	0.234	-	0.710	0.008	*0.099*	0.181	0.038
Neuropathy	-	-	-	-	1	2	-	1	-	-
	-	-	-	-	0.019	0.118	-	1.000	-	-
Paralysis	-	-	-	-	-	1	1	2	-	1
	-	-	-	-	-	1.000	1.000	1.000	-	0.543
DC	-	-	2	-	-	6	5	5	-	4
	-	-	0.259	-	-	0.404	0.399	0.227	-	0.312
Cerebellar Syndrome	-	-	-	-	-	-	1	-	-	1
	-	-	-	-	-	-	0.311	-	-	0.227
Seizure	-	-	1	-	-	2	1	4	-	2
	-	-	0.403	-	-	1.000	1.000	1.000	-	0.379
IS	-	-	-	1	-	1	1	6	-	1
	-	-	-	0.302	-	0.505	0.497	0.816	-	1.000

**HSV-1:** Herpes simplex virus-1; **HSV-2:** Herpes simplex virus-2; **VZV:** Varicella-Zoster virus; **EBV:** Epstein-Barr virus; **HCMV:** human cytomegalovirus; **HHV-6:** human herpesvirus 6; **EV:** Non-polio enterovirus; **DENV:** dengue virus; **YFV:** yellow fever virus; **ZIKV:** Zika virus; **IDH:** Initial diagnosis hypothesis; **DC:** Disorder of consciousness; **IS:** Infectious screening/sepsis; **IH:** Intracranial hypertension; **GBS:** Guillain Barré Syndrome; **ND:** Neurodegenerative disease. *P Values indicate differences between positive and negative patients for virus detection. P<0.05 was considered statistically significant.

Table 5 shows the correlation between positive PCR results in CSF and the signs and symptoms presented by the patients. 

**TABLE 5:** Correlation between Positive PCR results in CSF with Signs and Symptoms presented by Patients.

	HSV-1	HSV-2	VZV	EBV	HCMV	HHV-6	EV	DENV	YFV	ZIKV
Signs/Symptoms (n)*										
Headache (n=62)	1	2	4	2	-	17	30	28	1	10
Fever (n=47)	-	1	4	2	-	15	10	17	-	7
Conjunctivitis (n=3)	-	-	-	-	-	-	1	-	-	2
Retro-orbital pain (n=13)	-	-	1	-	-	3	2	4	-	5
Optical symptoms (n=36)	-	-	-	1	-	3	8	17	1	7
Prostration (n=6)	-	-	1	1	-	1	1	3	1	-
Myalgia (n=6)	1	1	-	-	-	1	1	2	-	1
Vomiting/Nausea (n=30)	-	1	4	-	-	5	7	13	-	6
Arthralgia (n=2)	-	-	-	-	-	-	-	2	-	1
Diarrhea (n=10)	-	-	1	-	-	3	2	5	-	3
Pruritus (n=1)	-	-	-	1	-	1	-	-	-	-
Edema (n=8)	-	-	1	1	-	1	-	4	-	2
Dizziness (n=12)	-	-	-	-	-	4	1	6	-	2
Dyspnea (n=4)	-	-	-	-	-	-	3	1	-	2
Cardiac symptom (n=12)	-	-	-	-	-	1	1	7	1	4
Respiratory symptom (n=24)	-	-	1	-	-	3	5	11	-	8
Irritability (n=10)	1	1	1	1	-	1	-	4	-	3
Dysphagia (n=9)	-	-	-	-	-	2	3	3	1	3
SW (n=15)	1	1	2	-	-	5	1	7	1	1
Tremors (n=11)	-	-	1	1	-	3	2	5	1	2
Diplopia (n=8)	-	-	1	-	-	2	1	2	-	3
Stiff neck (n=13)	1	1	1	-	-	7	2	3	1	1
Paresthesia (n=15)	-	-	-	-	1	4	3	7	1	1
Dysarthria (n=5)	-	-	-	-	-	2	2	1	1	1
Blurred vision (n=24)	-	-	-	1	-	3	7	7	1	6
Dysphonia (n=3)	-	-	-	-	-	1	1	1	-	-
Weakness LL (n=18)	1	1	1	-	-	5	7	12	1	4
Weakness UL (n=27)	1	1	2	-	-	3	5	5	1	4
Facial paresis (n=5)	-	-	-	-	-	1	-	3	-	1
Sleepiness (n=24)	-	-	1	2	-	7	4	9	1	3
Hyperreflexia (n=3)	-	-	-	1	-	1	-	2	-	-
Arreflexia (n=5)	-	-	-	-	-	2	1	1	-	1
Coma (n=6)	-	-	-	2	-	-	1	2	1	1
Death (n=5)	-	-	-	1	-	1	-	3	-	1
Hospitalization (n=83)	1	2	2	2	-	21	18	36	1	13
Immunodepression (n=44)	-	-	-	2	-	9	10	21	-	11
ATB (n=56)	-	1	4	2	-	15	10	21	-	11
ATV (n=14)	-	-	2	-	-	5	3	4	-	4

**HSV-1:** Herpes simplex virus-1; **HSV-2:** Herpes simplex virus-2; **VZV:** Varicella-Zoster virus; **EBV:** Epstein-Barr virus; **HCMV:** human cytomegalovirus; **HHV-6:** human herpesvirus 6; **EV:** Non-polioenterovirus; **DENV:** dengue virus; **YFV:** yellow fever virus; **ZIKV:** Zika virus; **LL:** Lower limbs; **UL:** Upper Limbs; **SW:** Symmetrical weakness; **ATB:** Antibiotics; **ATV:** Antiviral. *More than one signs/symptoms can occur in the same patient.

Five patients with viruses detected in their CSF samples died: a 78-year-old female patient with DENV+ was hospitalized (2 days) with confusion and urinary tract infection, mobility difficulties, and facial paresis; a 60-year-old male patient presented with headache and DENV+ on radiological treatment of squamous cell carcinoma (immunocompromised); a 52-year-old female patient was immunocompromised, had fever (39ºC) and EBV+, was hospitalized with confusion and drowsiness and coma, evaluated as an inconclusive infectious screening, and died of sepsis; a 63-year-old female patient presented with meningoencephalitis was immunocompromised due to a kidney transplant (17 years ago) had fever peaks (39ºC), lack of appetite, confusion, diarrhea, bradycardia, and renal failure, tested positive for two viruses, HHV-6 and DENV; and a 34-year-old male patient with HIV prior to the episode was ZIKV+, had heartburn, weakness, and respiratory failure and was hospitalized due to severe lowering of consciousness, fever, vomiting, and diarrhea. 

## DISCUSSION

This descriptive study investigated the possible etiological agents associated with acute neurological syndromes in 420 patients by analyzing their CSF specimens, focusing on a possible viral etiology, and avoiding other microbiological agents. Applying different molecular tests enabled the evaluation of different viruses circulating in the Campinas region, which is responsible for different outcomes in terms of neurological manifestations. 

PCR and its adaptation to distinct applications have become relevant tools for acquiring infectious disease diagnoses. Studies point out that Gram stains, in meningitis cases, are inconclusive in 70% of cases^15^.

The identification of any pathogen in a sterile specimen, such as CSF, has a deleterious effect. The application of techniques able to detect nucleic acids in CSF has a reduced chance of false-positive results due to the lack of PCR inhibitors and the specificity of the assay, there is a decreased chance of doubt regarding infection^15^. 

We found 420 samples without a specific diagnosis by traditional microbiological culture, with 148/420 (35.2%) samples being a reservoir of viral agents. The absence of information about the etiological agent can lead to an undesirable situation in which the treatment and conduct applied was not the most appropriate. This surveillance could be extrapolated since approximately 65% of the analyzed samples lacked an identified agent, an index similar to that in other studies. These negative results could be caused by other organisms - even different viruses not included in this study - or by some limitations inherent to the methods adopted. NAAT sensitivity depends on the viral load, and the timing of CSF sample collection can influence the results. CSF sampling takes a certain amount of time between the patient seeking care (and patients usually seek care after the onset of the most critical symptoms) and the material being collected and processed, resulting in possible clearance of the viral load by the time the specimen is analyzed, leading to false-negative results^1^ ^,^ ^3^ ^,^ ^15^. 

Comparing patients with positive results and those with no investigated viruses detected showed no statistical difference in protein, glucose, RBC count, or WBC count, showing the low predictive value of viral infections. Protein and glucose concentrations usually follow a normal ratio or even slightly increase with viral infections. Regarding cell count, whether lymphocytes tend to predominate the typical ratio may vary depending on the neurological syndrome. However, studies have shown alterations in leukocytes and high protein levels in about 48% of cases in contrast to those observed^7^ ^,^ ^31^.

Arboviruses have gained notoriety worldwide, especially after recent outbreaks. However, these viruses are endemic in economically developing countries such as Brazil. Among these viruses, 67/148 (45.2%) cases of DENV, 21/148 (14.2%) cases of ZIKV, and 3/148 cases of YFV (2%) were detected in the CSF samples^32^ ^-^ ^34^. 

DENV, the most abundant virus found in the evaluated samples, was present in almost half of the positive samples, representing 16.0% of all patients (67/420). Remarkably, among the different cases, there were no characteristic symptoms or manifestations. Thus, the absence of a characteristic that could be considered pathognomonic or statistically biased highlights the need to monitor this pluripotent virus. Headache was the most frequent manifestation, with 28/67 (41.8%) of the patients having this complaint, followed by fever (which was not always present or observed) and optical symptoms, which were observed in 17/67 (25.4%). However, 3/67 (4.5%) patients had encephalopathy, which exposes 60% of all cases of encephalopathy included in this study (p = 0.031)^32^ ^-^ ^34^. 

Since the notorious outbreak of ZIKV in 2015, extensive research has investigated congenital microcephaly related to this virus. However, adults present a spectrum of manifestations, including neurological complications. ZIKV was detected in 21/148 (14.2%) of all positive cases. Remarkable symptoms were observed, mostly with respect to respiratory and optical complications. Optical manifestations were present, indicating the importance of ZIKV in these complications. The only patient who died of ZIKV had pulmonary sepsis as well as HIV^35^.

There were 3/420 (0.7%) cases of YFV, two of which were caused by encephalitis. This low frequency, 0.7%, was similar to that of another study in the same country during a similar period using serum samples. This may be related to the reemergence of YFV and the outbreak of wild virus circulation in 2016. It is worth mentioning that this virus also circulates in other South American countries, leading to constant surveillance of a possible new outbreak. It is important to highlight that, although there was a large vaccination campaign against the virus in this period due to the increase in cases of probable sylvatic origin and since the vaccination may be associated with some symptoms, the vaccination was registered in none of the cases^36^_._

EV is one of the main viral etiologic agents related to acute viral neurological infection and widely associated with meningitis worldwide. In this study, 20.3% (30/148) of the positive cases tested positive. EV tends to be the most prevalent virus associated with NS; however, in this study, DENV was the most frequently detected virus. This does not reduce the high frequency of EV; rather, it reinforces the relevance of DENV in the Campinas region. The symptoms described for these cases are diverse; in this study, important symptoms associated with headache were observed in 100% of the cases of EV, a finding similar to that reported by Soares et al^40^. EV was present in the CSF samples from patients diagnosed with neuritis in 6/30 cases (20.0%, p = 0.008). This shows the importance of this finding since these five cases represent 38.4% of the cases of this manifestation, showing, together with ZIKV, relevance in the viral investigation of ophthalmic diseases. GBS was also identified in four patients, one with DENV-1 (mentioned above), an atypical condition, but with precedents in the literature on HHV^2^ ^,^ ^3^ ^,^ ^13^ ^,^ ^36^ ^-^ ^40^.

HSV-1 and HSV-2 are ubiquitous viruses worldwide. In our study, HSV-1 was detected in only one sample, which also contained HSV-2. This lack of detection - although both viruses have high seroprevalence in the population - has already been exposed, being detected in cohorts of patients younger than 15 years. VZV was identified with other viruses in one case with HHV-6+EV, the only detection of three pathogens in the same CSF sample. VZV has also been identified in ZIKV. CNS VZV infection tends to be self-limiting and is predominant in children. Reactivation of VZV in the absence of rash and skin lesions is known as *zoster sine herpete*, so VZV should be considered a potential cause of meningitis even without these injuries^41^ ^,^ ^42^.

EBV was identified in 4/148 (2.7%) samples testing positive for viral infections, a rate lower than that reported by Bastos et al.. al., with a prevalence of 23.7% of cases of EBV in patients older than 15 years but 1.5% higher than that reported in a study in Spain (1.2%). More than 30% of the cases of myelitis with the detected virus had EBV in their material (1/3, p = 0.074). One of these patients died during the hospitalization. Thus, in this population, its detection can present a very worrisome prognosis^3^ ^,^ ^43^.

HCMV is a virus closely related to complex conditions; however, in our study, only one patient was infected with it. Previous studies by our group found more cases using the same diagnostic method, although in other patients at another time. A study performed by Ory et al. analyzed 566 samples and found that four (1.6%) patients tested positive for HCMV. Several factors may be related to this reduced number as well as to other HHVs, but it is complex to say. The variations may be due to different groupings (which may include socioeconomic differences), the absence of active virus replication, or even methodological failure. HHV latency and the high serological prevalence found with this viral family do not reflect the active viral infection; therefore, the detection of latent particles can also influence the higher prevalence in some studies^2^ ^,^ ^3^ ^,^ ^21^ ^,^ ^43^ ^,^ ^44^.

HHV-6 was the most prevalent HHV in this study, with 32/148 (21.6%) among the positive cases for any virus, an unusual finding: some studies have an index below 1% when they focus on patients with encephalitis and meningitis exclusively. The relationship between this virus and encephalitis is well described in the literature; however, in this study, the most common clinical hypothesis was meningitis, with 9/32 cases (28.1%), a statistically significant difference (p = 0.034). It was detected in 2/10 (20%) cases of encephalitis and viral infection. Headache was the most prevalent manifestation in patients with HHV-6, at 17/32 (53.1%)^41^ ^,^ ^45^.

Some viruses, although included in this study, were not detected in the CSF by the methods used. Another study focusing on investigating viruses in Brazil also could not find ROCV, ILHV, WNV, SLEV, WEEV, EEEV, VEEV, and MAYV. In our study, CHIKV was not detected in CSF samples. Some studies have provided evidence of the circulation of these viruses in recent years in Brazil, and epidemiological surveillance should be encouraged in developing a plan for managing a potential future outbreak^3^ ^,^ ^46^ ^-^ ^48^. 

Molecular biology, with the application of NAAT, can provide tools of fundamental importance for obtaining an accurate diagnosis. Thus, viral surveillance should be encouraged and fostered to monitor the circulation of viral species. In countries such as Brazil, which have several endemic viruses, this surveillance must be constant. Finally, we were able to identify several viral species circulating in the region of Campinas, Brazil, providing information about some of their manifestations. Therefore, future studies should monitor the circulation of these viruses, analyze the symptomatologic pattern, and relate the findings of viral genomes with other species. 
